# Hypoxia acts as an environmental cue for the human tissue-resident memory T cell differentiation program

**DOI:** 10.1172/jci.insight.138970

**Published:** 2021-05-24

**Authors:** Farah Hasan, Yulun Chiu, Rebecca M. Shaw, Junmei Wang, Cassian Yee

**Affiliations:** 1 Department of Melanoma Medical Oncology, University of Texas (UT) MD Anderson Cancer Center, Houston, Texas, USA.; 2 MD Anderson Cancer Center UTHealth Graduate School of Biomedical Sciences, Houston, Texas, USA.; 3 Department of Immunology, UT MD Anderson Cancer Center, Houston, Texas, USA.

**Keywords:** Immunology, Hypoxia, T cells

## Abstract

Tissue-resident memory T cells (T_RM_) provide frontline defense against infectious diseases and contribute to antitumor immunity; however, aside from the necessity of TGF-β, knowledge regarding T_RM_-inductive cues remains incomplete, particularly for human cells. Oxygen tension is an environmental cue that distinguishes peripheral tissues from the circulation, and here, we demonstrate that differentiation of human CD8^+^ T cells in the presence of hypoxia and TGF-β1 led to the development of a T_RM_ phenotype, characterized by a greater than 5-fold increase in CD69^+^CD103^+^ cells expressing human T_RM_ hallmarks and enrichment for endogenous human T_RM_ gene signatures, including increased adhesion molecule expression and decreased expression of genes involved in recirculation. Hypoxia and TGF-β1 synergized to produce a significantly larger population of T_RM_ phenotype cells than either condition alone, and comparison of these cells from the individual and combination conditions revealed distinct phenotypic and transcriptional profiles, indicating a programming response to milieu rather than a mere expansion. Our findings identify a likely previously unreported cue for the T_RM_ differentiation program and can enable facile generation of human T_RM_ phenotype cells in vitro for basic studies and translational applications such as adoptive cellular therapy.

## Introduction

Tissue-resident memory T cells (T_RM_) are a recently defined subset of non-recirculating memory T cells that reside in peripheral tissues and are important in frontline defense against viral infections and associated with antitumor immunity. However, despite intense study, relatively little is known regarding T_RM_ differentiation. Studies in mice have shown CD8^+^ T_RM_ are seeded by early effector T cells that differentiate into resident memory in situ, likely in response to local cues (1, 2). These cues are thought to differ by tissue but remain poorly defined. TGF-β is known to be critical in T_RM_ establishment, and attempts to identify other resident phenotype inductive factors have focused on cytokines (1–9). Studies using mouse splenocytes 4 to 5 days postactivation have shown that TNF-α and IL-33 synergize with TGF-β to induce an intestinal resident T cell phenotype (CD103^+^Ly6C^−^CD69^+^) as well as downregulation of the transcription factor Kruppel-like factor 2 (KLF2) and its target genes sphingosine-1 phosphate receptor 1 (*S1PR1*) and *SELL* (CD62L), which is required for T_RM_ establishment (4, 10).

Oxygen tension is another factor that distinguishes peripheral tissues from the circulatory environment: typical physiological oxygen concentrations encountered by T cells can be as high as 10% to 12.5% in arterial blood and 3% to 6% in most healthy tissues and as low as 0.5% to 6% in the spleen and lymph nodes (11–13). Notably, T_RM_ were first described in barrier tissues, which are known to be relatively hypoxic (1, 14–18). In addition, any area of local inflammation can become hypoxic because of the increased density of metabolically active cells (19, 20). It has also been shown that oxygen consumption by activated neutrophils is sufficient to cause microenvironmental hypoxia (21). Thus, it is conceivable that the change in oxygen tension experienced by T cells in peripheral tissues and local inflammation could serve as a cue for the tissue residency program. We hypothesized that hypoxia may contribute to a TGF-β1–induced T_RM_ phenotype in human CD8^+^ T cells.

To date, the majority of studies on T_RM_ have been conducted in mice as human studies are subject to more technical and regulatory constraints. Although there is considerable overlap between the phenotypes reported for mouse and human T_RM_, there also appear to be key differences, notably illustrated by the differing importance of Hobit in T_RM_ differentiation (8, 22–24). Such differences highlight the necessity for studying T_RM_ differentiation in human cells, both to further understanding of basic molecular mechanisms and to facilitate effective translational application. Here, we demonstrate that human T cells differentiated during exposure to hypoxia and TGF-β1 develop a T_RM_ phenotype characterized by expression of protein markers and a transcriptional profile reminiscent of endogenous T_RM_.

## Results

### Human CD8^+^ T cells differentiated in hypoxia and TGF-β1 acquire a T_RM_ phenotype.

The necessity of TGF-β in T_RM_ formation is well documented, but additional cues are required, as TGF-β alone is not sufficient to induce T_RM_ phenotype (4, 8). Previous studies in mouse splenocytes demonstrated a role for inflammatory cytokines in T_RM_ phenotype induction when used in combination with TGF-β (4, 10). Given the relative hypoxia in inflamed tissues, we postulated that low oxygen tension could provide additional cues for T_RM_ differentiation. To determine whether hypoxia can contribute to induction of a T_RM_ phenotype, we sorted naive (CD45RA^+^CCR7^+^) CD8^+^ T cells from human peripheral blood mononuclear cells (PBMCs), activated them for 4 days under hypoxia (2% O_2_) or normal cell culture conditions (~20% O_2_) to generate “early effectors,” and then cultured an additional 2 days in the presence of TGF-β1 (25).

**Figure 1:**
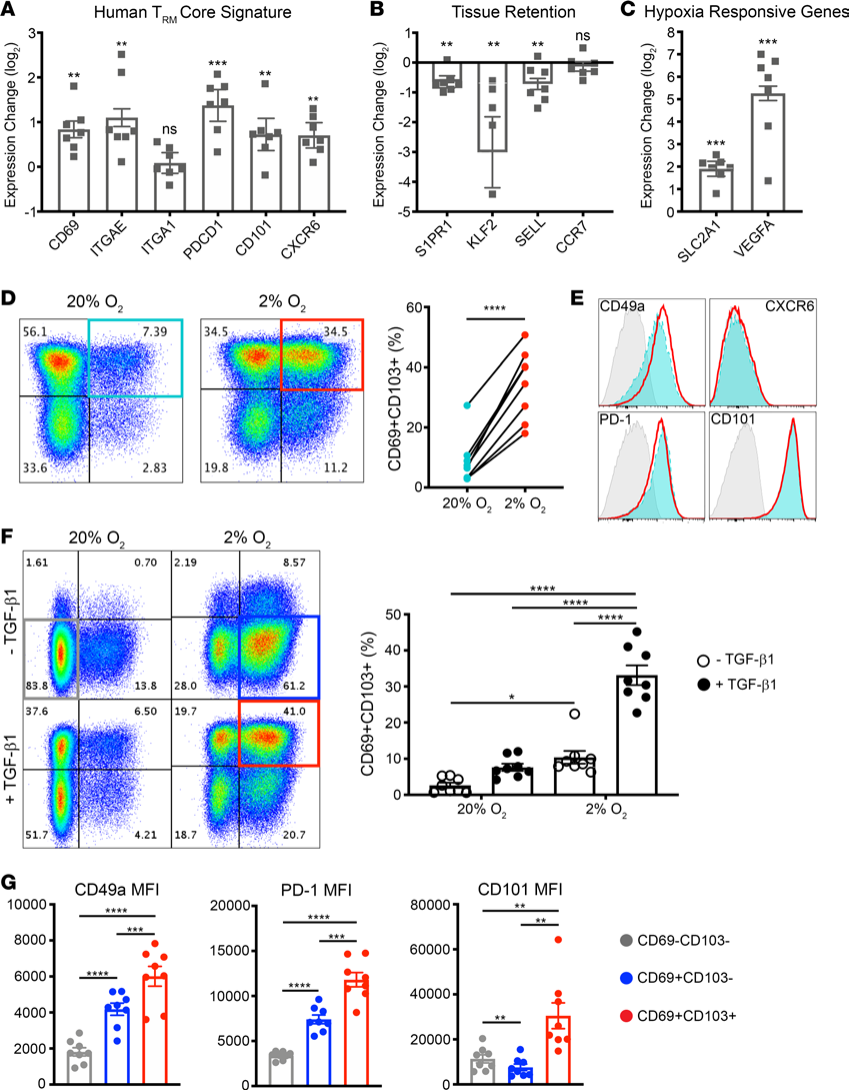
Hypoxia and TGF-β1 synergize to induce a CD69^+^CD103^+^ population that expresses human T_RM_-associated markers. Naive CD8^+^ T cells sorted from PBMCs were activated in 20% O_2_ (AtmosO_2_) or 2% O_2_ (hypoxia) for 4 days and then for an additional 2 days with the addition of recombinant human TGF-β1 (rhTGF-β1). Expression levels of T_RM_-associated genes were analyzed via quantitative real-time PCR. (**A**–**C**) Fold change of gene transcript levels in 2% O_2_ + TGF-β1 over 20% O_2_ + TGF-β1. (**D**) The frequency of the CD69^+^CD103^+^ T_RM_-like population and (**E**) expression of T_RM_-associated markers were assessed by flow cytometry. Representative results from 1 donor are shown (**D** and **E**). Blue histograms represent CD69^+^CD103^+^ cells from 20% O_2_ + TGF-β1, red histograms represent CD69^+^CD103^+^ cells from 2% O_2_ + TGF-β1, and gray histograms represent fluorescence minus one (FMO). (**A**–**C**) *n* = 7, 3 independent experiments; paired *t* test with Benjamini, Krieger, and Yekutieli correction for multiple comparisons; **q < 0.01, ***q < 0.001, ****q < 0.0001; FDR < 0.05, data are mean ± SEM. (**D**) *n* = 8, 3 independent experiments; ratio paired *t* test; ****P < 0.0001. Naive CD8^+^ T cells were activated in 20% O_2_ or 2% O_2_ for 4 days and then for an additional 2 days with or without rhTGF-β1. (**F**) Frequency of the CD69^+^CD103^+^ population and (**G**) expression of T_RM_-associated markers on CD69^–^CD103^–^ cells (20% O_2_), CD69^+^CD103^–^ cells (2% O_2_), and CD69^+^CD103^+^ cells (2% O_2_ + TGF-β1) were assessed by flow cytometry; representative pseudocolor plots shown for 1 donor, *n* = 8. Two-way ANOVA (**F**) or repeated measures 1-way ANOVA (**G**), *P adj < 0.05, **P adj < 0.01, ***P adj < 0.001, ****P adj < 0.0001; data are mean ± SEM.

We used quantitative real-time PCR (qPCR) to assess the bulk populations for expression of a panel of T_RM_-associated genes. Cells differentiated in 2% O_2_ + TGF-β1 showed upregulation of most of the genes Kumar et al. recently identified as the human T_RM_ core signature, namely *CD69*, *ITGAE* (CD103), *PDCD1* (programmed cell death 1 [PD-1]), *CD101*, and *CXCR6* (Figure 1A and ref. 22). We did not observe a difference in *ITGA1* (CD49a) transcript levels. In addition, transcripts of genes important in T cell recirculation (*S1PR1*, *KLF2*, *SELL*) were downregulated, further suggesting a residency program (Figure 1B). The downregulation of *S1PR1* and *KLF2* was previously shown to be critical to murine T_RM_ differentiation, and decreased levels of these genes have also been observed in endogenous human T_RM_ (10, 22, 23, 26). Elevated expression of the canonical hypoxia-responsive genes *SLC2A1* and *VEGFA* confirmed the cells were responding to hypoxic conditions (Figure 1C). Together, these results indicate that when human CD8^+^ T cells are differentiated under hypoxia in combination with TGF-β1, they acquire a T_RM_-like transcriptional profile.

We then evaluated protein-level expression of the core human T_RM_ signature via flow cytometry (22). In all donors tested there was an increase in CD69^+^CD103^+^ cells in the 2% O_2_ + TGF-β1 condition compared with 20% O_2_ + TGF-β1 (Figure 1D). Notably, unlike a previous report in mice, circulating naive human CD8^+^ T cells did not appreciably express CD103 (ref. 27 and Supplemental Figure 1B; supplemental material available online with this article; https://doi.org/10.1172/jci.insight.138970DS1). Cell viability was comparable or better in 2% O_2_ + TGF-β1 versus 20% O_2_ + TGF-β1 (Supplemental Figure 1C). These CD69^+^CD103^+^ cells expressed CD49a, PD-1, and CD101; however, we did not observe CXCR6 surface protein expression despite transcriptional upregulation in the bulk population (Figure 1, A and E). Although CD69 and CD103 expression is commonly used to define T_RM_, contention remains regarding which of these markers best denotes tissue residency, partly due to their heterogeneous expression in endogenous T_RM_ (28). Thus, we compared expression levels of the T_RM_-associated markers CD49a, PD-1, and CD101 among the CD69^–^CD103^+^, CD69^+^CD103^+^, and CD69^+^CD103^–^ populations from the 2% O_2_ + TGF-β1 condition. As expected, the CD69^+^CD103^+^ population had the highest levels of PD-1 and CD101 surface expression (Supplemental Figure 1D). CD49a expression was also high but not significantly different from levels observed in the CD69^+^CD103^–^ population. In comparing the 2 oxygen conditions, we found the most dramatic increase in population fold change to be in the CD69^+^CD103^+^ population (Supplemental Figure 1, E and F). Based on these results, we focused further analysis on the CD69^+^CD103^+^ population as our in vitro induced T_RM_ cells (hereafter i-T_RM_).

### Hypoxia and TGF-β1 are synergistic cues for T_RM_ phenotype acquisition.

Since the atmospheric oxygen level of normal tissue culture conditions is higher than what T cells experience in vivo, we evaluated the effect of 10% O_2_, which represents a physiologically relevant, nonhypoxic oxygen level in the circulation (11). Although there was a slight increase in CD69^+^CD103^+^ T cells in 10% O_2_ + TGF-β1 compared with 20% O_2_ + TGF-β1, correcting for multiple comparisons showed no significant differences in T_RM_-associated gene expression between the 2 conditions (Supplemental Figure 2, A–C). In addition, the fold increase of the CD69^+^CD103^+^ population over 20% O_2_ + TGF-β1 was significantly greater for the 2% O_2_ + TGF-β1 condition versus 10% O_2_ + TGF-β1 (Supplemental Figure 2D).

To assess the individual contributions of hypoxia and TGF-β1 to generation of T_RM_ phenotype cells, we performed in vitro differentiation experiments in 2% or 20% O_2_ with or without TGF-β1. Hypoxia primarily induced CD69^+^ cells whereas TGF-β1 induced CD103^+^ cells, in congruence with reports that hypoxia and TGF-β can drive expression of these markers, respectively (29–31). Although hypoxia or TGF-β1 alone induced a modest population of CD69^+^CD103^+^ cells, their combination appeared to synergize induction of T_RM_ phenotype to levels markedly greater than the additive effects of either condition alone (Figure 1F). The CD69^+^CD103^+^ cells induced by hypoxia + TGF-β1 expressed the highest levels of CD49a, PD-1, and CD101, compared with the majority populations in the 20% O_2_ and 2% O_2_ conditions (CD69^–^CD103^–^ and CD69^+^CD103^–^, respectively) (Figure 1G).

### In vitro induced T_RM_ exhibit enrichment for endogenous human T_RM_ gene signatures.

**Figure 2:**
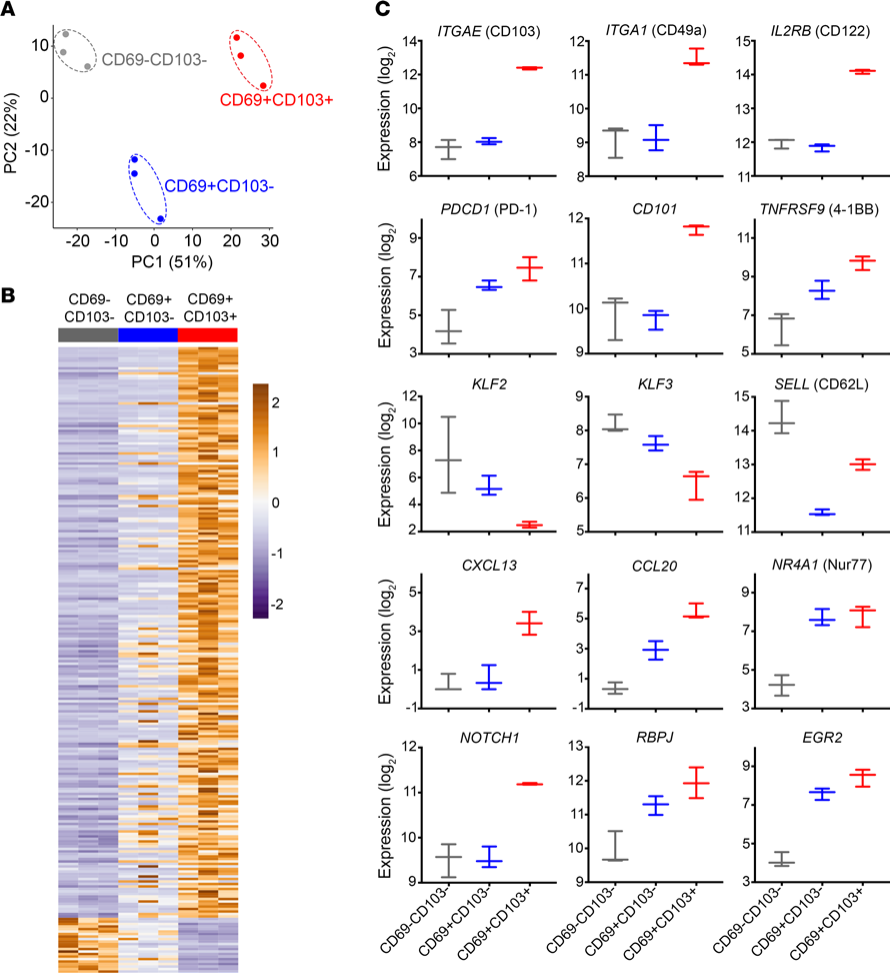
Hypoxia + TGF-β1 induces transcriptional changes similar to those reported for endogenous T_RM_. CD69^–^CD103^–^ (20% O_2_), CD69^+^CD103^–^ (2% O_2_), and CD69^+^CD103^+^ (2% O_2_ + TGF-β1) CD8^+^ T cells were generated as described earlier and sorted before RNA isolation and transcriptome analysis via RNA sequencing (RNA-Seq) (*n* = 3). (**A**) Principal component analysis (PCA) of paired CD69^–^CD103^–^, CD69^+^CD103^–^, and CD69^+^CD103^+^ CD8^+^ T cells based on the global transcriptome. (**B**) Heatmap showing the top 250 differentially expressed genes (DEGs) for CD69^–^CD103^–^ (gray), CD69^+^CD103^–^ (blue), and CD69^+^CD103^+^ (red) cells generated in 20% O_2_, 2% O_2_, and 2% O_2_ + TGF-β1, respectively. Legend represents *z* score, orange color indicates upregulation and purple color indicates downregulation. Differential expression determined by |log_2_FC| ≥ 1 and FDR < 0.05. (**C**) Normalized expression levels of selected differentially expressed T_RM_-associated genes, showing min, median, and max. TNFRSF9, 4-1BB; RBPJ, recombination signal binding protein for immunoglobulin kappa J region; EGR2, early growth response 2.

Since differences in T_RM_ marker expression among the CD69^–^CD103^–^ (20% O_2_), CD69^+^CD103^–^ (2% O_2_), and CD69^+^CD103^+^ (2% O_2_ + TGF-β1) cells suggested that these represent distinct populations, each was sorted and transcriptionally profiled via RNA sequencing (RNA-Seq). Principal component analysis (PCA) confirmed that these 3 populations were distinct (Figure 2A). Hierarchical clustering showed distinct gene signatures for CD69^–^CD103^–^ and CD69^+^CD103^+^ cells, whereas CD69^+^CD103^–^ cells had a somewhat intermediate transcriptional profile (Figure 2, B and C). Comparison of the top DEGs between CD69^+^CD103^+^ and CD69^–^CD103^–^ cells revealed gene expression patterns consistent with those reported for endogenous human T_RM_, including increased expression of *ITGAE*, *EGR2*, granulysin (*GNLY*), interleukin 2 receptor subunit β (*IL2RB)*, Bcl2 modifying factor (*BMF*), RasGEF domain family member 1B (*RASGEF1B*), and nuclear receptor subfamily 4 group A member 1 (*NR4A1*), and decreased expression of *SELL*, *KLF2*, and *KLF3*, indicating a non-recirculating transcriptional program (Figure 2C; Supplemental Table 1; and refs. 22, 23, 32). *S1PR1*, a KLF2 target gene, was downregulated 1.5-fold (*P adj* < 0.05), but this did not meet our fold change threshold. CD69^+^CD103^+^ cells demonstrated increased expression of *ITGA1*, *PDCD1*, *CD101*, and *TNFRSF9*, all of which are consistently reported as upregulated in endogenous human T_RM_ (22, 23, 26, 33). We also observed elevated transcripts encoding the transcription factor NOTCH1, which is known to contribute to maintenance of lung T_RM_, and *RBPJ*, which is central to Notch signaling (Figure 2C and refs. 23, 34).

T_RM_ often express various chemokines, perhaps as part of their “alarm function,” to recruit immune cells to local tissues (35). Consistent with the endogenous T_RM_ profile, i-T_RM_ upregulated *CXCL13* and *CCL20*, as well as *CCL4*, *CCL5*, and *CCL22* (Figure 2C; Supplemental Table 1; and refs. 22, 23, 26). Changes in expression of genes that have currently undefined roles but are consistently reported in endogenous human T_RM_ were also observed, such as upregulation of *MYO7A* and *RGS1* and downregulation of *SERPINE2*, *RAP1GAP2*, and *RASGRP2* (Supplemental Table 1 and refs. 22, 23, 26).

**Figure 3:**
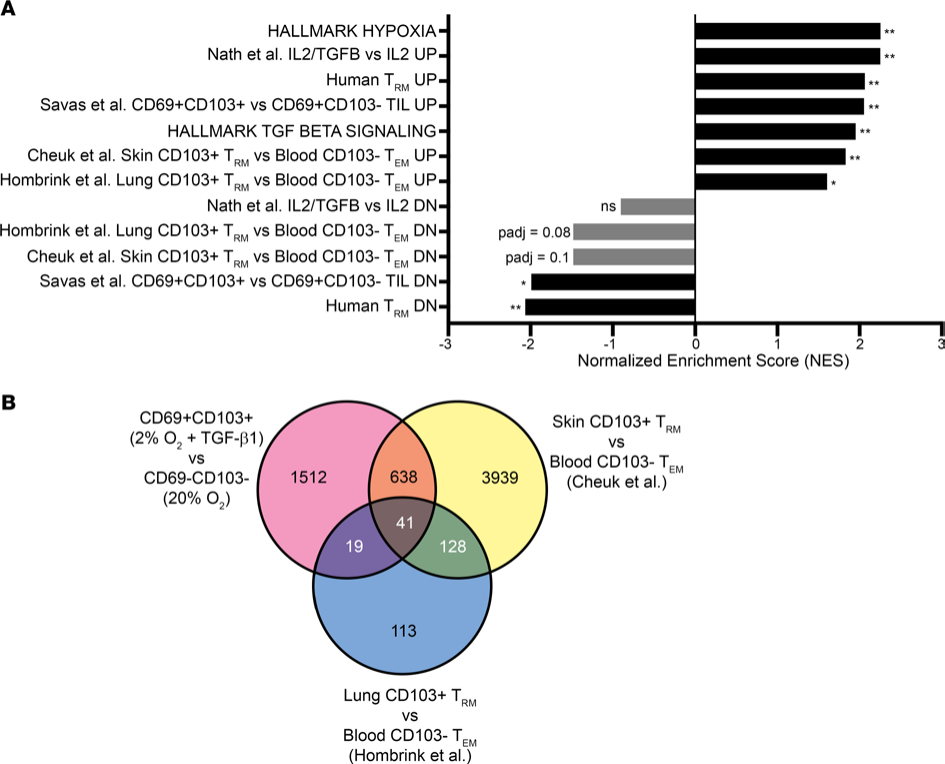
The hypoxia + TGF-β1 i-T_RM_ transcriptional profile is enriched for endogenous human T_RM_ gene signatures. CD69^–^CD103^–^ (20% O_2_), CD69^+^CD103^–^ (2% O_2_), and CD69^+^CD103^+^ (2% O_2_ + TGF-β1) CD8^+^ T cells were generated as described earlier and sorted before RNA isolation and transcriptome analysis via RNA-Seq (*n* = 3). (**A**) GSEA of relevant gene signatures derived from endogenous T_RM_ and resident memory-like tumor-infiltrating lymphocytes (TIL_RM_) in the transcriptome of CD69^+^CD103^+^ versus CD69^–^CD103^–^, presented as normalized enrichment score (NES), *P adj < 0.05, **P adj < 0.01. (**B**) Venn diagram showing the overlap of DEGs between CD69^+^CD103^+^ and CD69^–^CD103^–^ cells and gene sets derived from endogenous human skin (Cheuk et al., ref. 36) and lung (Hombrink et al., ref. 23) T_RM_.

To gain insight into the physiological relevance of our findings, we performed gene set enrichment analysis (GSEA) using gene signatures from published data sets of endogenous human CD103^+^ T_RM_ compared with CD103^–^ effector memory T cells (T_EM_) from peripheral blood (23, 36). We observed that the transcriptional profile of CD69^+^CD103^+^ cells versus CD69^–^CD103^–^ cells was similar to those of T_RM_ from human skin (Cheuk et al., ref. 36) and lung (Hombrink et al., ref. 23) tissues versus blood T_EM_ (Figure 3A). Signatures of genes upregulated in T_RM_ were more enriched, reflecting that increases in gene expression predominated the transcriptional differences between CD69^+^CD103^+^ and CD69^–^CD103^–^ cells, with enrichment driven by genes such as *ITGAE*, *ITGA1*, *EGR2*, *BMF*, *PERP*, *CCL20*, *NOTCH1*, *RBPJ*, and *DUSP4*, among others (Figure 2B, Figure 3A, and Supplemental Table 2). Analysis of these data sets for overlap revealed that all 3 shared genes related to TGF-β, cell adhesion, motility, and migration and genes encoding regulators of T cell differentiation: *EGR2* and *NR3C1* (glucocorticoid receptor, Figure 3B, and refs. 37, 38). Importantly, *NR3C1* has previously been shown to promote the formation of memory precursor T cells and can be upregulated by hypoxia (38, 39). In addition, there was considerable overlap between the transcriptional profiles of our i-T_RM_ and human skin T_RM_, including a number of canonical hypoxia-responsive genes (e.g., *LDHA*, *MMP9*, *CA9*), as well as *LGALS3*, a newly identified marker of human skin T_RM_ (40).

Multiple recent profiles of TILs in various solid tumor types have reported the presence of CD103^+^ TIL_RM_ (41–45). Therefore, we compared CD69^+^CD103^+^ cells (hypoxia + TGF-β1) with CD69^+^CD103^–^ cells (hypoxia alone) or CD69^–^CD103^–^ cells (normal culture conditions) and observed enrichment of the CD8^+^CD69^+^CD103^+^ human breast cancer TIL signature reported by Savas et al. (Figure 3A, Supplemental Figure 3, and ref. 41). As hypoxia and TGF-β are common features of the tumor microenvironment (TME), our results suggest they may contribute to CD103^+^ TIL generation in vivo. Additionally, our results may help explain recent observations that TIL_RM_ are more radioresistant, as hypoxia and TGF-β are known to contribute to radioresistance, and DEGs in radioresistant human skin T_RM_ show enrichment in the hypoxia pathway, relative to tissue-infiltrating T cells (40, 46–48).

Since the immunosuppressive TME tends to drive T cells toward exhaustion, it is possible that cells differentiated in hypoxia + TGF-β1 could become exhausted. Despite reports that hypoxia and TGF-β1 can cause T cell exhaustion, we did not observe differential expression of the exhaustion-associated genes *ENTPD1* (CD39), *HAVCR2* (TIM3), *LAG3*, *CTLA4*, *CD38*, *SLAMF6*, *CD244*, *TIGIT*, and *TOX* (Supplemental Table 1 and refs. 49, 50). To further compare our i-T_RM_ with exhausted T cells, we performed GSEA using published human T cell exhaustion signatures. The Quigley et al. signature showed an inverse enrichment pattern; that is, genes that are downregulated in exhausted T cells were upregulated (positively enriched) in our i-T_RM_ (Supplemental Figure 4A and ref. 51). Genes that are upregulated in exhausted T cells showed a trend toward negative enrichment in our data set but did not reach statistical significance. The CD39^+^ exhaustion signature of upregulated genes (Gupta et al.) was enriched in our data set; however, aside from *PDCD1*, the shared genes driving enrichment were not associated with exhaustion and instead encoded transcription factors and enzymes spanning a broad array of biological functions (Supplemental Table 2 and ref. 52). In comparison, genes upregulated in T_RM_ from the skin of healthy individuals (Cheuk et al.) were more enriched in our data set, in terms of both significance and proportion (Supplemental Figure 4B and ref. 36). These data show that, compared with cells from normal cell culture conditions, i-T_RM_ do not exhibit an exhausted transcriptional profile.

### Pathway analysis reveals biological commonalities between in vitro induced T_RM_ and endogenous human T_RM_.

**Figure 4:**
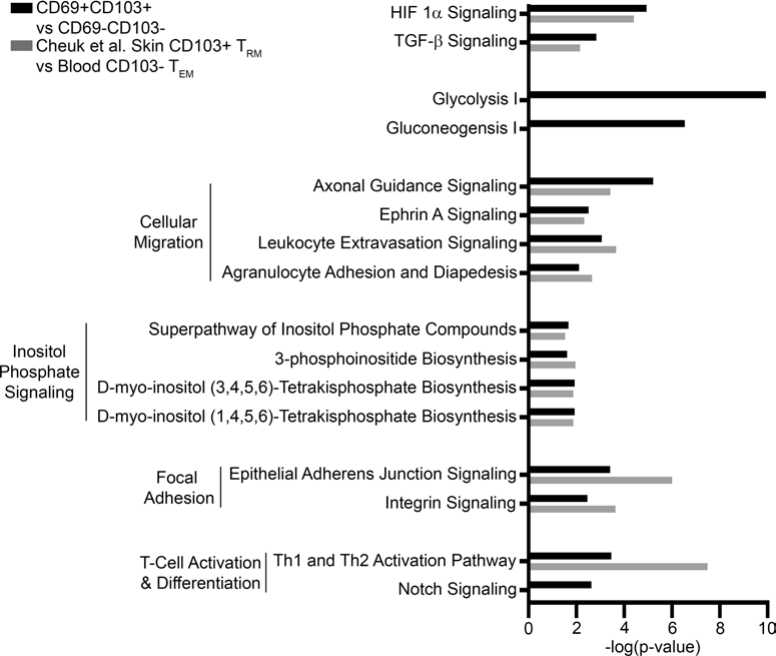
Pathways involved in metabolism, migration, and T_RM_ generation and maintenance are differentially regulated in hypoxia + TGF-β1 i-T_RM_. Differentially regulated IPA canonical pathways in hypoxia + TGF-β1 i-T_RM_ relative to their expression in CD69^–^CD103^–^ cells and endogenous human skin T_RM_ relative to blood T_EM_ (Cheuk et al., ref. 36), shown with negative-log-transformed *P* values. HIF-1α, hypoxia-inducible factor 1α.

Ingenuity Pathway Analysis (IPA; QIAGEN) comparing CD69^+^CD103^+^ cells with CD69^–^CD103^–^ cells revealed that many of the DEGs were in the glycolysis and gluconeogenesis pathways (Figure 4). Given that hypoxia is a major regulator of cellular metabolism, these results were expected. There was also an enrichment of genes in the Notch signaling pathway, which has been reported in human lung T_RM_ (23). To better understand the functional relevance of the hypoxia + TGF-β–induced T_RM_ transcriptional profile, we ran pathway analysis on the Cheuk et al. data set and found that many of the same biological pathways were differentially regulated. Mirroring our earlier overlap analysis, we observed enrichment of DEGs in the HIF-1α and TGF-β signaling pathways in both transcriptional profiles (Figure 4). Changes in leukocyte extravasation signaling, agranulocyte adhesion and diapedesis, epithelial adherens junction signaling, and integrin signaling pathways, all involved in focal adhesion and related to changes in migratory programming, were common to their endogenous T_RM_ and our i-T_RM_. Multiple pathways involved in inositol phosphate signaling were also enriched, in agreement with a previous report that PI3K signaling is implicated in cytokine-induced downregulation of *KLF2* and may play a role in generation of T_RM_ in vivo (10). Remarkably, the axonal guidance pathway was also differentially regulated in both analyses. Axonal guidance, while at first seemingly unrelated to T_RM_ and unreported in current T_RM_ literature, is a process whereby environmental cues influence cell migratory patterns (53). Many of the same factors governing axon guidance are also known to regulate immune cell trafficking and can be regulated by hypoxia and/or TGF-β (53–59). Overall, these results show that our i-T_RM_ display striking similarities to endogenous human T_RM_ at the biological pathway level.

### In vitro induced T_RM_ maintain their phenotype in response to milieu.

**Figure 5:**
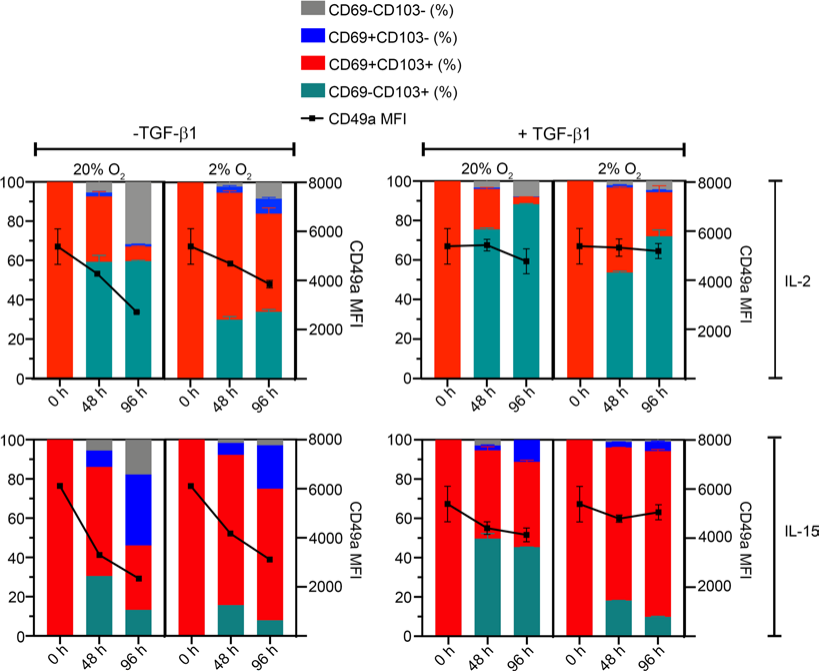
In vitro induced T_RM_ maintain their phenotype in response to environmental cues. CD69^+^CD103^+^ CD8^+^ T cells were generated in 2% O_2_ + TGF-β1 as described earlier, sorted, and distributed into parallel cultures in 20% O_2_ or 2% O_2_ in the presence of IL-2 or IL-15, with or without TGF-β1. Cells were analyzed for expression of T_RM_-associated markers via flow cytometry at 0 hours, 48 hours, and 96 hours (indicated along the *x* axis). Data are from 2 independent donors (*n* = 2) and 2 independent experiments, excepting the IL-15 without TGF-β1 condition (*n* = 1); mean ± SEM shown.

To assess the stability of our i-T_RM_ phenotype, we sorted CD69^+^CD103^+^ cells generated using hypoxia + TGF-β1 and cultured them in parallel in 20% O_2_ or 2% O_2_, with IL-2 or IL-15, and with or without TGF-β. After 48 hours of culture at 20% O_2_ with IL-2 and without TGF-β1, a slight majority of cells had lost CD69 expression (becoming CD69^–^CD103^+^) with a gradual decrease in CD49a expression; however, culture in hypoxia helped maintain CD69 expression, consistent with our earlier results (Figure 5). Culture with IL-2 and TGF-β1 at both oxygen levels seemed to drive the cells toward CD103 single positivity but also helped maintain CD49a expression. In contrast, culture with IL-15 enabled maintenance of CD69 expression that was further enhanced in hypoxia, although CD49a expression declined. Culture in hypoxia with IL-15 and TGF-β1 maintained expression of CD69, CD103, and CD49a in the majority of cells. IL-2 is the prototypical cytokine for effector T cell culture, whereas IL-15 is important for the maintenance of memory T cells and the development and/or maintenance of T_RM_ in several organs (2, 24, 60–62). The dependence of our i-T_RM_ on IL-15 may be related to their increased expression of *IL2RB*, which encodes the β chain of the IL-15 receptor (CD122), in line with murine studies on the importance of IL-15 responsiveness for skin T_RM_ (3, 62). Further, it has been shown that exposure of human PBMCs to IL-15 and TGF-β1 can also induce a CD69^+^CD103^+^ phenotype in CD8^+^ T cells (8). Thus, these data demonstrate that in addition to TGF-β1, hypoxia can cooperate with other environmental cues, such as IL-15, to promote a T_RM_ phenotype in T cells. Our results also indicate that maintenance of the i-T_RM_ phenotype relies on environmental cues, mirroring the plasticity and environmental adaptability of endogenous (murine) T_RM_, which can alter their phenotype but reacquire T_RM_ characteristics when exposed to the appropriate milieu (63, 64).

### Hypoxia and TGF-β1 influence the human T_RM_ differentiation program.

**Figure 6:**
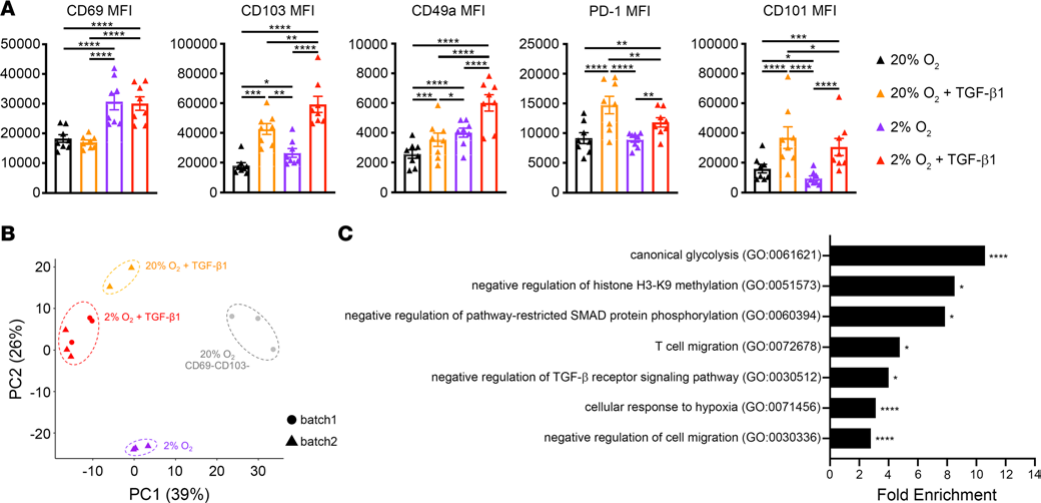
CD69^+^CD103^+^ cells generated in single and combination conditions are distinct. Naive CD8^+^ T cells were activated in 20% O_2_ or 2% O_2_ for 4 days and then for an additional 2 days with or without TGF-β1. (**A**) Expression of T_RM_-associated markers was assessed by flow cytometry, *n* = 8. Two-way ANOVA, *P adj < 0.05, **P adj < 0.01, ***P adj < 0.001, ****P adj < 0.0001; data are mean ± SEM. CD69^+^CD103^+^ cells generated in 20% O_2_ + TGF-β1, 2% O_2_, or 2% O_2_ + TGF-β1 were sorted before isolation of RNA for analysis via mRNA sequencing (*n* = 2–3). (**B**) PCA of paired CD69^+^CD103^+^ cells from the different culture conditions (batch2) and CD69^–^CD103^–^ cells from previous analysis (batch1). (**C**) Selected significantly overrepresented Gene Ontology (GO) terms with fold enrichment, *P adj < 0.05, ****P adj < 0.0001.

We showed earlier that hypoxia or TGF-β1 alone can generate CD69^+^CD103^+^ cells, but their combination results in the greatest induction (Figure 1F). This observation raised the question of whether CD69^+^CD013+ cells from the combination condition are simply greater in number or actually phenotypically distinct. Comparison of T_RM_ core signature protein expression via flow cytometry indicated that CD69^+^CD103^+^ populations from the different conditions are not identical. Consistent with previous results, CD69^+^CD103^+^ cells generated in hypoxia displayed higher CD69 expression, and those that were exposed to TGF-β1 were marked by higher CD103 expression (Figure 6A). TGF-β1 exposure was also related to higher expression of PD-1 and CD101. Remarkably, hypoxia alone induced elevated CD103 and CD49a expression; however, the combination of hypoxia and TGF-β1 resulted in CD69^+^CD103^+^ cells with the highest levels of CD103 and CD49a expression (Figure 6A). While the influence of TGF-β1 on CD103 and CD49a has been reported, to our knowledge, this is the first implication of hypoxia in induction of these canonical T_RM_-associated integrins (5, 30, 31, 65). These observations also further support our findings that hypoxia and TGF-β1 are synergistic cues for T_RM_ phenotype acquisition.

To compare the populations at the transcriptional level, we sorted CD69^+^CD103^+^ cells generated by hypoxia alone (2% O_2_), TGF-β1 alone (20% O_2_ + TGF-β1), and their combination (2% O_2_ + TGF-β1) and conducted RNA-Seq. PCA showed that all 3 groups of CD69^+^CD103^+^ cells clustered away from CD69^–^CD103^–^ cells from normal cell culture conditions analyzed previously (20% O_2_, batch1), indicating relative similarity (Figure 6B). All data were normalized and corrected for batch variation, and we validated that this clustering pattern was not due to batch effects by including CD69^+^CD103^+^ samples from our previous analysis (batch1), confirming that they clustered with the 2% O_2_ + TGF-β1 group samples as expected. Cells exposed to TGF-β1 clustered closer to each other than to those cultured in hypoxia alone, suggesting that the effects of TGF-β1 predominate in our system. Differential expression analysis was performed only among samples from the same batch (batch2) in order to analyze matched samples. Gene Ontology (GO) analysis on the 1014 DEGs between the 3 group conditions (*P adj* < 0.001, determined by likelihood ratio test) revealed enrichment of genes involved in glycolysis and hypoxia response, regulation of TGF-β signaling (TGF-β receptor and SMADs), and cell migration (Figure 6C). Interestingly, genes encoding regulators of histone methylation were also enriched, suggesting an epigenetic component.

**Figure 7:**
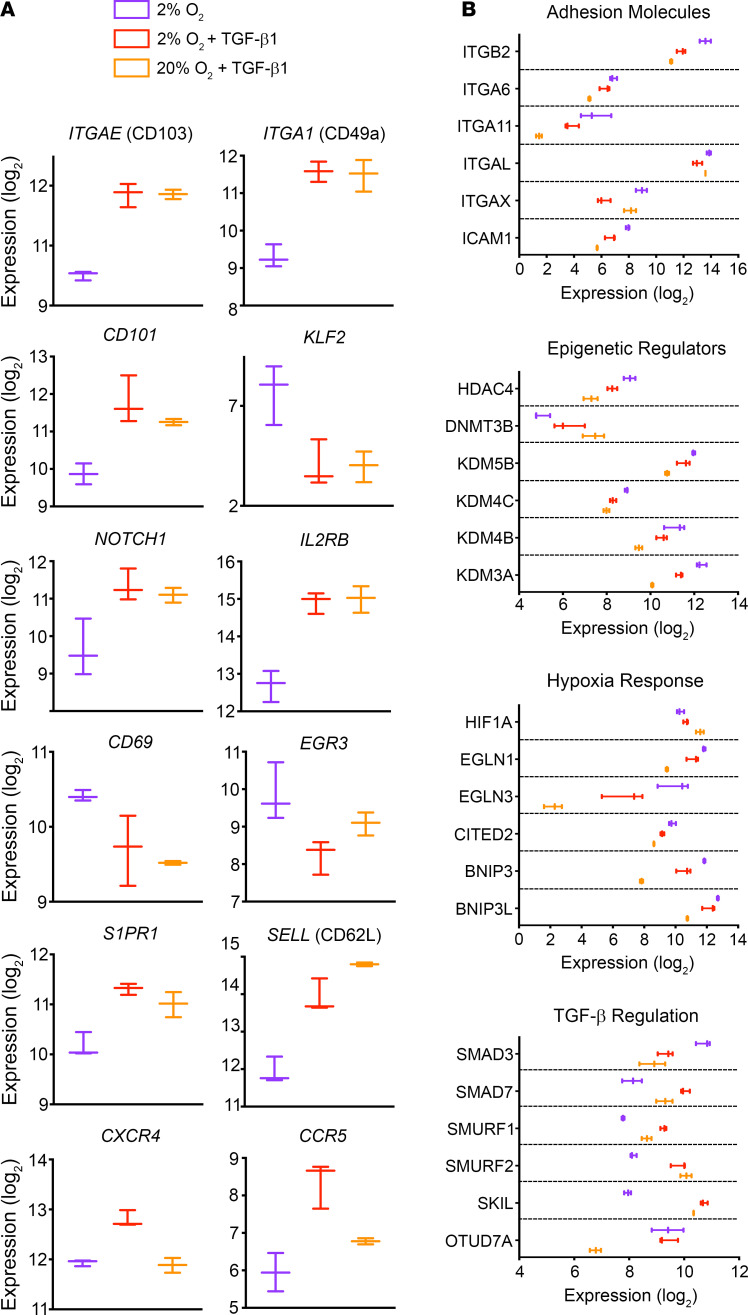
Transcriptional comparison of CD69^+^CD103^+^ cells generated in single and combination conditions. CD69^+^CD103^+^ cells generated in 20% O_2_ + TGF-β1, 2% O_2_, or 2% O_2_ + TGF-β1 were sorted before RNA isolation and analysis via mRNA sequencing (*n* = 2–3). (**A**) Normalized expression levels of selected differentially expressed T_RM_-associated genes, showing min, median, and max. (**B**) Normalized expression levels of selected DEGs derived from GO terms in Figure 6C, grouped by category. Differential expression determined by likelihood ratio test and *P adj* < 0.001. EGLN1, egl-9 family hypoxia inducible factor 1; CITED2, Cbp/p300 interacting transactivator with Glu/Asp rich carboxy-terminal domain 2; BNIP3, BCL2 interacting protein 3; SMURF1, SMAD specific E3 ubiquitin protein ligase 1; SKIL, SKI like proto-oncogene.

Closer examination of T_RM_ signature genes revealed many that were primarily influenced by TGF-β1, including *ITGAE*, *CD101*, *KLF2*, *NOTCH1*, and *IL2RB* (Figure 7A). *ITGA1* transcript levels were also similar between TGF-β1–treated groups, suggesting a posttranscriptional mechanism for the CD49a upregulation observed (Figure 6A and Figure 7A). Expression of *CD69*, *S1PR1*, *SELL*, and *EGR3* was more heavily influenced by hypoxia, and *CXCR4* and *CCR5* transcript levels were highest only in CD69^+^CD103^+^ cells from the combination condition (Figure 7A). Examination of the genes driving enrichment in the GO analysis showed that hypoxia exposure also influenced expression of genes encoding a number of adhesion molecules and epigenetic regulators, including upregulation of several histone lysine demethylases (KDMs): *KDM5B*, *KDM4C*, *KDM4B*, and *KDM3A* (Figure 7B). Expression patterns of genes involved in hypoxia response and regulation of TGF-β signaling provided some insight into the interaction of these 2 pathways. TGF-β1 exposure upregulated *HIF1A*, which encodes the transcription factor HIF-1α, and downregulated several genes encoding negative regulators of HIF-1α: *EGLN1*, *EGLN3*, and *CITED2* (66, 67). Hypoxia exposure resulted in increased expression of *BNIP3* and *BNIP3L*, which was slightly dampened by TGF-β1 exposure. Apart from their roles in apoptosis, BNIP3 and BNIP3L also mediate autophagy, which is involved in generation and perhaps maintenance of memory T cells, such as human liver T_RM_, and this is the function we propose, given our earlier observation that cellular viability is not compromised by hypoxia (68–70). Similarly, hypoxia increased expression of *SMAD3*, which encodes an important TGF-β signaling mediator, and decreased expression of several negative regulators of TGF-β signaling (*SMAD7*, *SMURF1*, *SMURF2*, *SKIL*). However, cells exposed to hypoxia + TGF-β had higher levels of *SMAD7*, *SMURF1*, and *SKIL* transcripts. These observations illustrate how hypoxia and TGF-β might potentiate and attenuate each other’s signaling and activity in a complex molecular network promoting the T_RM_ differentiation program.

### Hypoxia-induced T_RM_ phenotype is partially recapitulated by HIF stabilization.

**Figure 8:**
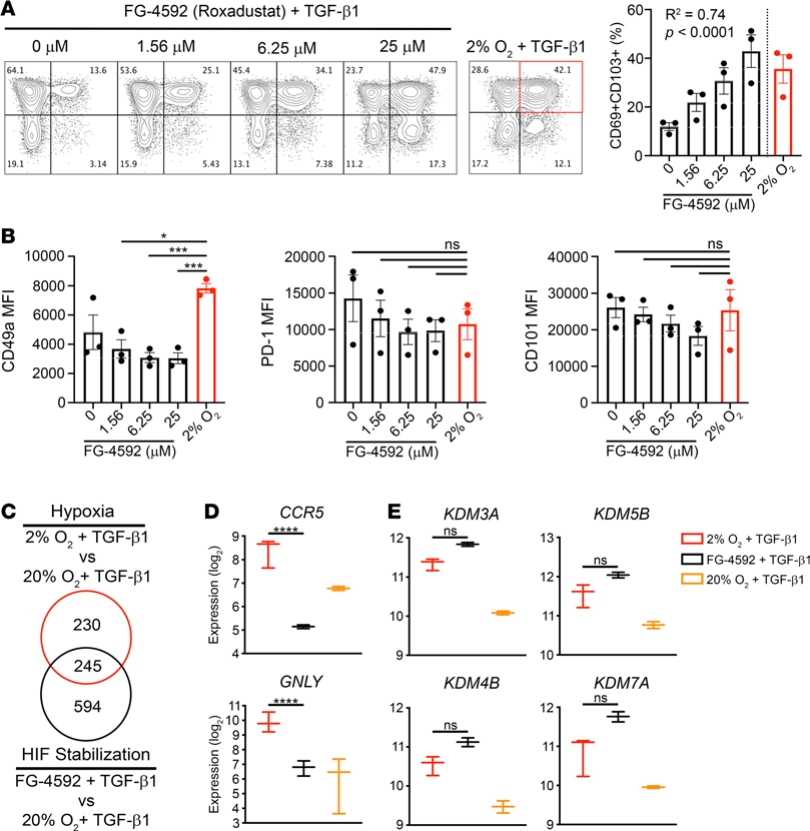
HIF stabilization partially reproduces hypoxia-induced T_RM_ phenotype. Naive CD8^+^ T cells were activated in 20% O_2_ (AtmosO_2_) in the presence of FG-4592 for 4 days and then for an additional 2 days with TGF-β1. Cells activated in 2% O_2_ with addition of TGF-β1 on day 4 are shown in red for comparison. (**A**) Frequency of the CD69^+^CD103^+^ population and (**B**) expression of T_RM_-associated markers by the CD69^+^CD103^+^ population were assessed by flow cytometry, with representative results shown in **A** for 1 donor, *n* = 3. Repeated measures 1-way ANOVA (**A** and **B**) followed by test for trend (**A**) or Dunnett’s multiple comparisons test (**B**), *P adj < 0.05, ***P adj < 0.001; data are mean ± SEM. CD69^+^CD103^+^ cells generated in FG-4592 or 2% O_2_, with addition of TGF-β1 were sorted before RNA isolation and analysis via mRNA sequencing (*n* = 2–3), with differential expression determined by |log_2_FC| ≥ 1 and FDR < 0.05. (**C**) Venn diagram showing the overlap of DEGs between CD69^+^CD103^+^ cells generated via FG-4592 or 2% O_2_ relative to 20% O_2_ (all with addition of TGF-β1). (**D**) Normalized expression levels of selected DEGs between CD69^+^CD103^+^ cells generated in FG-4592 or 2% O_2_, showing min, median, and max; ****P adj < 0.0001. CD69^+^CD103^+^ cells generated via TGF-β1 alone (20% O_2_) are shown for reference.

HIFs are transcription factors governing the cellular response to hypoxia that are regulated by prolyl hydroxylase (PHD) enzymes, which require oxygen to function (71). To assess whether HIFs mediated the hypoxic induction of CD69^+^CD103^+^ cells, we used the HIF PHD inhibitor FG-4592 (roxadustat) to stabilize HIFs during differentiation experiments in 20% O_2_ with the addition of TGF-β1 (72). We observed a dose-dependent increase in CD69^+^CD103^+^ cells that reached frequencies similar to those in 2% O_2_ + TGF-β1 (Figure 8A). This increase was primarily driven by increased CD69 expression, consistent with a recent report showing *CD69* is a HIF target gene (Supplemental Figure 5 and ref. 29). To further assess whether HIF stabilization by PHD inhibition reproduces the hypoxia-induced T_RM_ phenotype, we compared expression of CD49a, PD-1, and CD101 on CD69^+^CD103^+^ cells generated via both routes. Although the addition of FG-4592 slightly decreased CD49a, PD-1, and CD101 expression when compared with TGF-β1 alone (0 μM), PD-1 and CD101 expression levels were not significantly different from those of cells differentiated in hypoxia + TGF-β1, suggesting that HIFs are involved in T_RM_ phenotype acquisition (Figure 8B). HIF stabilization via PHD inhibition did not recapitulate CD49a expression; however, this may be due to off-target effects of FG-4592. PHDs also regulate collagen biosynthesis, and since CD49a is part of the collagen-binding complex very late antigen-1, its expression may be affected by PHD inhibition (73).

To gain insight into the contributions of hypoxia versus HIFs, we compared the transcriptional profiles of CD69^+^CD103^+^ cells induced by 2% O_2_ + TGF-β1 or FG-4592 + TGF-β1, using CD69^+^CD103^+^ cells induced by TGF-β1 alone as a common reference. While the DEGs did not completely overlap, there was considerable similarity (Figure 8C). T_RM_-associated genes generally were not among the DEGs, again likely owing to the dominant effect of TGF-β1. Direct comparison of CD69^+^CD103^+^cells induced by hypoxia or FG-4592 showed there was no significant difference in their expression of core T_RM_ signature genes; however, genes encoding CCR5 and *GNLY* were upregulated only by hypoxia (Figure 8D). Interestingly, genes encoding several of the KDMs discussed earlier were among the overlapping DEGs, and direct comparison of CD69^+^CD103^+^ cells induced by hypoxia or FG-4592 revealed comparable expression; however, the activity of these enzymes may differ between the 2 conditions (Figure 8E). These KDMs belong to a class of enzymes known as 2-oxoglutarate–dependent oxygenases, which utilize oxygen in their functions and thus are also regulated by oxygen in a HIF-independent manner (74). As this is the same class of enzymes to which HIF PHDs belong, it is possible that their activity would be affected by FG-4592; however, this is unlikely at the doses used here (73). These demethylases vary in their targets and sensitivity to oxygen and chemical inhibitors, so specific changes in activity and their downstream consequences in this study are currently unclear. In light of the large degree of overlap in expression of T_RM_-associated genes between the TGF-β1–treated groups, it is plausible that alterations in chromatin accessibility are partially responsible for the increased frequency of CD69^+^CD103^+^ cells in hypoxia and hypoxia-mimetic conditions. Indeed, recent work characterizing murine small intestine T_RM_ differentiation reported high *KDM5B* expression in a likely T_RM_ precursor population, concordant with the early time point used in our study (75). Overall, these results suggest that HIFs are involved in T_RM_ phenotype acquisition, with the possibility that HIF-independent effects of hypoxia may play a role as well.

## Discussion

The well-documented heterogeneity among T_RM_ is likely due to varying environments across tissue niches, and we propose that hypoxia is one of the many possible environmental cues for the T_RM_ program (6, 28, 32, 33, 36, 63, 76–78). The intestine, skin, and reproductive tract, sites with well-characterized T_RM_ populations, are known to be relatively hypoxic (14–16). This is further reflected in our findings of similarity between hypoxia-cultured i-T_RM_ and human skin T_RM_, due in part to the enrichment of hypoxia-responsive genes. However, questions may be raised regarding the relevance of hypoxia as an environmental cue in the lung, which, in addition to being an important site of T_RM_ accumulation, is the organ responsible for oxygen exchange. Interestingly, a study on human lung T_RM_ reported increased expression of transcripts encoding HIFs and enrichment of hypoxia-inducible genes when compared with T cells from peripheral blood (23). These findings could be attributed to the sources of tissue samples used: patients with lung tumors or chronic obstructive pulmonary disease, which are both conditions that can create hypoxic environments (79–81). However, although oxygen levels in the alveoli are high, oxygen tensions in lung tissue can be comparable to those in other organs (median ~5%; refs. 80, 82, 83). A recent study comparing murine lung airway and interstitial T_RM_ highlights how microenvironmental variation within the same organ can impact T_RM_ heterogeneity (76). Varying degrees of vascularization create a range of oxygen levels within an organ, presumably making the epidermis more hypoxic than the dermis of the skin and the white pulp more hypoxic than the red pulp of the spleen (12, 13, 84). Small T_RM_ populations have also been described in secondary lymphoid organs (SLOs), which are predominantly sites of recirculation (26, 85). In this case microenvironmental hypoxia could promote T_RM_ differentiation as a function of distance from blood vessels within the organ, or through inflammation-induced hypoxia, which is relevant to any inflamed tissue. Moreover, it is believed that some SLO T_RM_ are derived from emigrants of T_RM_ originally differentiated in nonlymphoid tissues (85). Microanatomical availability and receipt of additional signals, such as from TGF-β, would also influence residency programming. It is possible that hypoxia is not a universal driver of T_RM_ in all tissues, just as T_RM_ in different organs vary in their requirement for IL-15 (60). Sources of active TGF-β for T_RM_ differentiation may also differ between tissues: provided by keratinocytes in the skin, Kupffer cells in the liver, dendritic cells in lymphoid and other organs, and perhaps monocytes and/or macrophages. These cells express integrins necessary to release active TGF-β from the latent form present in the environment — either bound to extracellular matrix via latent TGF-β binding protein or anchored to immune cell membranes via glycoprotein-A repetitions predominant protein (GARP/LRRC32) or LRRC33 (27, 86–91).

Our findings also raise questions regarding the role of metabolism in T_RM_ differentiation. Hombrink et al. suggested that a major role of Notch signaling in lung T_RM_ is regulation of metabolic programs because inhibition of Notch signaling affected genes involved in glycolysis, oxidative phosphorylation, and fatty acid metabolism pathways (23). It has also been suggested that deletion of the purinergic receptor P2RX7, which is known to modulate glycolysis, impairs T_RM_ formation via metabolic dysregulation, as P2RX7-deficient cells displayed decreased mitochondrial mass and function, defective aerobic glycolysis, and impaired glucose uptake (92–94). While hypoxia reportedly promotes effector differentiation in T cells via HIF-driven glycolysis, TGF-β seems to be important for memory T cell formation, likely via effects on mitochondria (95–97). In contrast to hypoxia, few reports describe the role of TGF-β in T cell metabolism. It has been shown that TGF-β can increase mitochondrial membrane potential (a measure of mitochondrial activity) in T cells and that CD8^+^CD103^+^ tumor-specific T cells have increased spare respiratory capacity; however, the connection to oxidative phosphorylation and/or fatty acid metabolism is unclear and may also be dependent on T cell differentiation status (97–100). Interestingly, P2RX7 activity induces HIF-1α (likely involved in P2RX7’s regulation of T cell metabolism) as well as TGF-β receptor II, thereby increasing sensitivity to TGF-β and providing another possible avenue of integration of these pathways in endogenous T_RM_ (93, 97, 101).

How hypoxia and TGF-β synergize to induce CD69^+^CD103^+^ cells remains an open question, and our results indicate that HIF-mediated mechanisms are involved. HIFs are known to modulate several T_RM_ phenotype features, including upregulation of *CD69* and *TNFRSF9*, and downregulation of *SELL* and *S1PR1*, as reflected in our results (29, 95, 102, 103). Further, inhibition of HIF-1α/β complex formation by HIF-1β deletion in murine T cells maintained CD62L expression, causing their accumulation in lymph nodes and preventing migration to nonlymphoid tissues. (102). Our work and that of others suggests TGF-β may potentiate HIF activity by upregulating *HIF1A* and promoting HIF stabilization and activity via downregulation of the negative regulators PHD2 and CITED2 (104, 105). Notably, HIF-1α can also be induced by other stimuli reportedly involved in T_RM_ generation and/or maintenance, namely IL-15, TNF-α, and P2RX7 stimulation by extracellular ATP (101, 106, 107). Moreover, IL-15 and TNF-α can induce CD69 expression similarly to hypoxia (8, 108). Thus, it is unclear to what extent hypoxia is a relevant cue in vivo or whether hypoxia in our system mimics the effects induced by other inflammatory stimuli. However, the cooperation we observed between hypoxia and IL-15 suggests these cues are complementary rather than redundant. We also observed changes in expression of several epigenetic regulators, suggesting hypoxia-induced chromatin remodeling may alter accessibility to hypoxia-inducible transcription factors such as HIFs and the glucocorticoid receptor as well as TGF-β–inducible transcription factors such as SMAD3 and EGR2 (109). Future studies are warranted to further dissect the mechanism via which hypoxia and TGF-β synergize to induce the T_RM_ phenotype.

In recent years multiple reports have suggested that TIL_RM_ are important in antitumor immunity. Specifically, the accumulation of CD103^+^ TILs in a wide array of solid tumor malignancies, including breast, lung, ovarian, pancreatic, and melanoma, is associated with favorable prognosis (41–45). In addition, murine model studies have shown that skin T_RM_ are protective against melanoma (110, 111). Thus, there is much interest in leveraging resident phenotype T cells for immunotherapy; however, current approaches focus on using vaccination to induce these cells in vivo because endogenous T_RM_ are difficult to isolate and culture, and there is no reliable method to generate these cells in vitro (92, 112, 113). The method described in this study may enable in vitro generation of T_RM_ phenotype cells and has the potential for application to multiple existing adoptive cellular therapy (ACT) modalities. The elevated PD-1 expression on i-T_RM_ make them attractive for combination with PD-1 blockade. Indeed, a recent study in metastatic melanoma patients reported that CD103^+^ TILs significantly expanded during anti–PD-1 therapy (114). There is also potential for i-T_RM_ ACT to treat viral diseases: the importance of T_RM_ in antiviral immunity is well established, and a recent study found that elite HIV controllers have high magnitudes of HIV-specific T_RM_ in their lymphoid tissues, suggesting that these cells may control chronic viral infections (26). We show that even in unfavorable conditions (20% O_2_ with IL-2) i-T_RM_ can maintain their phenotype because by 48 hours approximately 30% of the cells remained CD69^+^CD103^+^. In addition, since preparation of ACT products for administration typically occurs within a 12-hour period, we believe the majority of i-T_RM_ would retain their phenotype during processing, as well as in vivo upon receiving suitable environmental cues. We are applying the findings of this study to expansion protocols for production of i-T_RM_ in sufficient quantity for therapeutic purposes.

To our knowledge this study is the first to recapitulate T_RM_ phenotype and transcriptional signature in vitro from human peripheral blood–derived T cells, as well as the first to identify hypoxia as a cue for the human T_RM_ differentiation program. While there are obvious limitations to experiments that can be conducted in humans, we believe the studies described provide compelling evidence that hypoxia is an environmental cue that can contribute to acquisition of a T_RM_ phenotype, supported by the observation that hypoxia + TGF-β i-T_RM_ recapitulate the transcriptional and proteomic landscape of endogenous T_RM_. These observations suggest that lower oxygen tensions such as those found in peripheral tissues, sites of inflammation, and tumors can promote the T_RM_ differentiation program. In vivo studies will be necessary to determine whether hypoxia and/or HIFs can contribute to T_RM_ differentiation in peripheral tissues and/or tumors and to determine whether i-T_RM_ can establish tissue residency after adoptive transfer.

## Methods

### Cell isolation and in vitro cell culture

Healthy donor PBMCs were collected via leukapheresis and stored in liquid nitrogen until use. CD8^+^ T cells were enriched from PBMCs using STEMCELL Technologies EasySep kits. Cells were then stained with fluorochrome-conjugated antibodies against CD8 (SK1), CD45RA (HI100), and CCR7 (G043H7) (all BioLegend, clones in parentheses). Naive CD8^+^CD45RA^+^CCR7^+^ T cells were sorted using a FACSAria IIIu or Fusion cell sorter (BD Biosciences). Sorted naive CD8^+^ T cells were resuspended in T cell culture media (RPMI, 10% FBS, l-glutamine, penicillin-streptomycin) with 10 IU/mL rhIL-2 (Prometheus) and equilibrated overnight to 2% O_2_ in a hypoxic chamber (Coy Laboratory Products) or in a standard cell culture incubator (~20% O_2_, Thermo Fisher Scientific). Cells were then activated with anti-CD3/anti-CD28 beads (Gibco, Thermo Fisher Scientific) at 3 cells per bead for 4 days. On day 4, 1.25 ng/mL rhTGF-β1 (BioLegend) was added, and cells were cultured for an additional 2 days. For phenotype stability assays i-T_RM_ were generated as described, sorted, and resuspended in media pre-equilibrated to 20% O_2_ or 2% O_2_ with 20 IU/mL rhIL-2 (Prometheus) or 10 ng/mL rhIL-15 (R&D Systems, Bio-Techne), with or without 1.25 ng/mL rhTGF-β1 (BioLegend).

### Flow cytometry

For analysis of human T_RM_-associated markers, beads were removed, and cells were washed once in staining buffer and stained with Live/Dead Fixable Aqua (Life Technologies, Thermo Fisher Scientific) and fluorochrome-conjugated antibodies against CD8 (SK1), CD69 (FN50), CD103 (ber-ACT8), PD-1 (EH12.2H7), CD101 (BB27), CXCR6 (K041E5), and CD49a (TS2/7) (all BioLegend, clones in parentheses). After staining, cells were fixed with Fixation Buffer (BioLegend) and stored in staining buffer until analysis. Stained cells were analyzed using an ACEA Novocyte 3000 flow cytometer. Single fluorochrome-stained compensation beads (UltraComp, eBioscience, Thermo Fisher Scientific) and FMO samples were used as controls. Data were analyzed using FlowJo software (BD Biosciences).

### qRT-PCR or qPCR

For analysis of human T_RM_-associated gene expression, cells were separated from beads and washed once in PBS. RNA was isolated using the QIAGEN RNeasy Plus Mini Kit. When necessary RNA was further purified/concentrated using the QIAGEN RNeasy MinElute Cleanup Kit. First-strand cDNA was synthesized using M-MLV Reverse Transcriptase (Thermo Fisher Scientific). qRT-PCR was performed using the QuantStudio 5 Real-Time PCR System and PowerUp SYBR Green Master Mix (Applied Biosystems, Thermo Fisher Scientific). Relative mRNA gene expression was normalized to the housekeeping gene *RPL13A*. Primers used are listed in Supplemental Table 3.

### RNA-Seq transcriptome analysis

Cells were sorted using a FACSAria IIIu cell sorter before RNA isolation using the RNeasy Plus Mini Kit followed by the RNeasy MinElute Cleanup Kit.

#### Regarding batch1.

The library was constructed using the Illumina TruSeq Stranded mRNA kit. RNA-Seq was conducted by the Sequencing and Non-Coding RNA Program of UT MD Anderson Cancer Center using the Illumina NextSeq500 platform. Raw reads were mapped to the *Homo sapiens* reference genome and transcriptome (GRCh38, GENCODEV23) by HISAT2 (version 2.1.0; ref. 115). Htseq-count (version 2.1.0) was used to get the counts for genes. R and Bioconductor packages DESeq2 (version 1.14.1) were used to identify DEGs (116). Genes (mRNA only, taking the protein-coding genes for *P* value adjustment) with FDR < 0.05 and |fold change| ≥ 2 were considered differentially expressed. R and Bioconductor package fgsea (version 1.10.0) was used to conduct GSEA. Gene sets were derived from several previously published data sets. The Nath et al. TGF-β signatures, Savas et al. TIL signatures, and Gupta et al. and Quigley et al. exhaustion signatures were derived from published differential expression tables (41, 51, 52, 117). The human T_RM_ signature was constructed from several studies (22, 23, 26, 36). For the lung and skin T_RM_ signatures, gene expression data were obtained from the Gene Expression Omnibus database (accessions GSE61397 and GSE83637) and analyzed using GEO2R or DESeq2, respectively (23, 36). FDR-adjusted *P* values less than 0.05 were considered significant. For analysis of intersection/overlap between gene sets, fold change thresholds were adjusted to ±1.5 and FDR ≤ 0.5 to account for the reduced sensitivity of microarray technology.

#### Regarding batch2.

The library was constructed using the NEBNext Ultra RNA Library Prep Kit for Illumina (New England Biolabs). mRNA sequencing was conducted by Novogene Corporation Inc. using the Illumina NovaSeq platform. Raw reads were mapped to the *Homo sapiens* reference genome and transcriptome (GRCh38, GENCODEV23) by HISAT2 (version: 2.1.0). Htseq-count (version: 2.1.0) was used to get the counts for genes. For analysis relevant to Figures 6 and 7: DEGs were determined using the likelihood ratio test function offered by Bioconductor R package DESeq2 (version 1.28.1). The genes (mRNA only, taking the protein-coding genes for *P* value adjustment) with *P adj* < 0.001 were selected as candidates. In order to decrease any sample-specific variation between the 2 batches of RNA-Seq data sets, supervised surrogate variable analysis using Bioconductor R package sva (version 3.36.0) based on the expression of human housekeeping genes (3804 genes) was performed (118, 119). For analysis relevant to Figure 8: R and Bioconductor package DESeq2 (version 1.28.1) was used to identify DEGs. Genes (mRNA only, taking the protein-coding genes for *P* value adjustment) with FDR ≤ 0.05 and |fold change| ≥ 2 were considered differentially expressed.

#### Regarding both batches.

Downstream logical and statistical outputs were generated using customized R scripts with packages dplyr (version 1.0.2), tidyr (version 1.1.2), tibble (version 3.0.4), stringi (version 1.5.3), tidyverse (version 1.3.0), clusterProfiler (version 3.16.1), and ggplot2 (version 3.3.2). Data have been deposited in the European Genome-phenome Archive (EGA) under the EGA ID/accession number EGAS00001005286.

#### Pathway analysis.

Functional analysis of significant DEGs (FDR < 0.05 and |fold change| > 2) was done with IPA software (version 60467501, QIAGEN) using all genes in the Ingenuity Knowledge Base as the reference set and right-tailed Fisher’s exact test in a core analysis to determine if pathways were significantly altered between conditions (−log_10_[*P* value] > 1.3).

#### GO.

Differentially expressed gene lists were tested for enrichment of GO pathways using the PANTHER classification system web-based gene list analysis tool, version 16.0 (120). The statistical overrepresentation test (released 20200728) was performed using the GO biological processes complete annotation data set and Fisher’s exact test with FDR < 0.05 correction for multiple testing.

### Statistics

Graphical presentation and statistical analysis of the data were performed using GraphPad Prism (Version 8). Data are displayed as mean ± SEM. Samples were matched/paired unless otherwise indicated. Results between experimental groups were compared using statistical tests described in the figure legends (*t* tests always 2-tailed and 1- or 2-way ANOVA always followed by Tukey’s multiple comparisons test, unless otherwise indicated). *P* < 0.05 was considered statistically significant.

### Study approval

All human sample collection was performed with informed consent and approved by the institutional review board of UT MD Anderson Cancer Center.

## Author contributions

FH conceptualized the study, designed and performed experiments, collected and analyzed data, and wrote the paper. YC analyzed RNA-Seq data. RMS performed experiments. JW analyzed data, provided intellectual contributions, and edited the paper. CY oversaw the study, provided intellectual contributions, and edited the paper.

## Supplementary Material

Supplemental data

Supplemental Table 1

Supplemental Table 2

Supplemental Table 3

## Version 1 05/24/2021

Electronic publication
