# Supplementary material for: Hypoxia acts as an environmental cue for the human tissue-resident memory T cell differentiation program
Source: JCI Insight. 2021 May 24;6(10):e138970. doi: 10.1172/jci.insight.138970 (PMC8262358; doi:10.1172/jci.insight.138970)
Supplement: Supplemental data [file jciinsight-6-138970-s195.pdf]

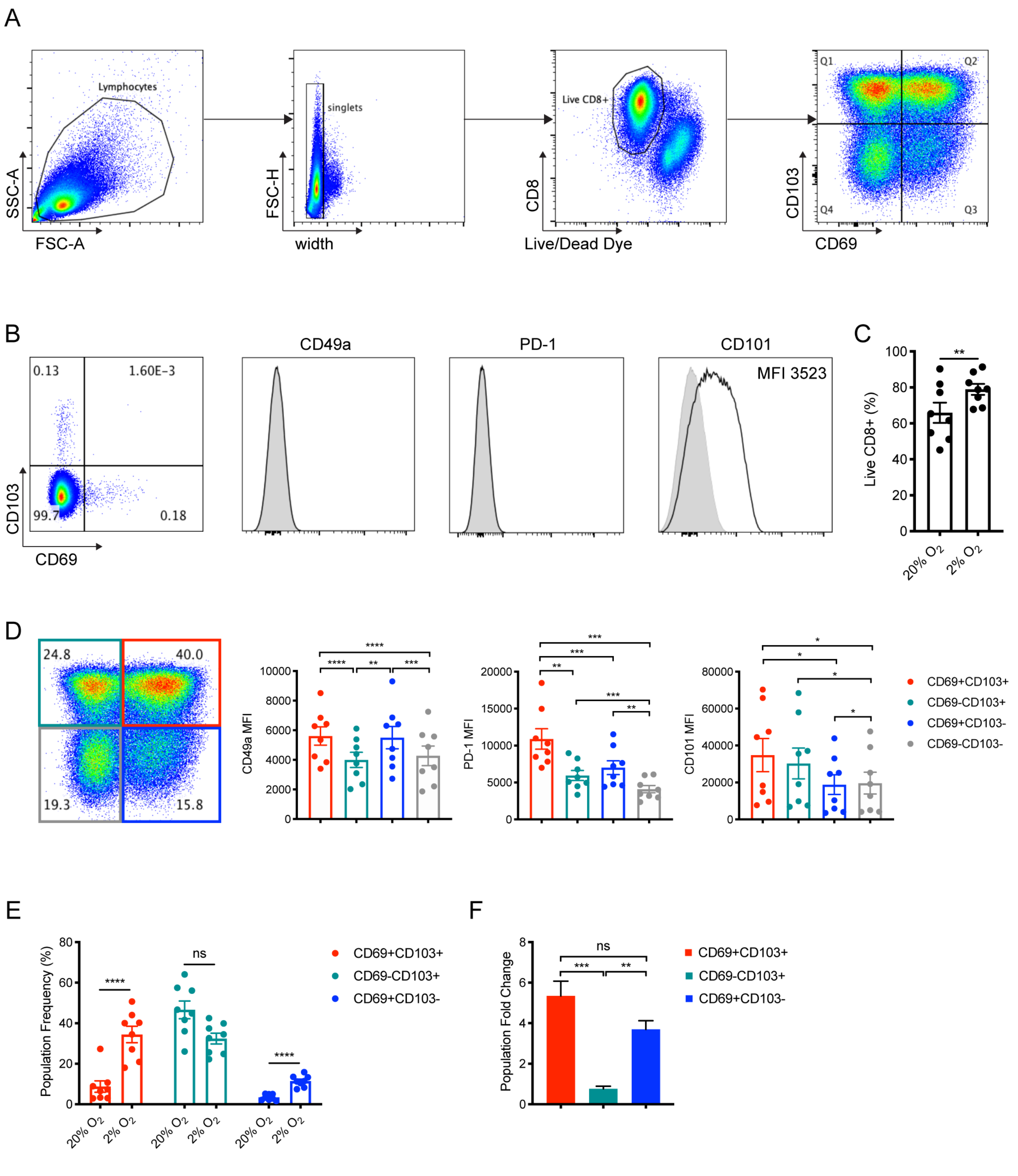

Supplemental Figure 1. Differentiation of human CD8+ T-cells in hypoxia and TGF-β1 results in induction of a CD69+CD103+ population. (**A**) Gating strategy used in flow cytometry analysis. (**B**) Naïve CD8+ T-cells were sorted from human PBMCs and assessed for expression of T_{RM}-associated markers by flow cytometry; gray histograms represent FMO. Naïve CD8+ T-cells were activated as described in Figure 1. (**C**) Cell viability determined by fixable viability dye (Invitrogen) in flow cytometry analysis and (**D**) expression of T_{RM}-associated markers was assessed by flow cytometry. (**E** and **F**) Changes in population frequencies comparing 20% O_2 + TGF-β1 and 2% O_2 + TGF-β1 conditions determined by flow cytometry. *n* = 8, 3 independent experiments; paired t-test (**C**), ratio paired t-test (**C**), or repeated measures one-way ANOVA followed by Tukey's (**D**) or Sidak's (**E** and **F**) multiple comparisons test; $^{*}P < 0.05$, $^{**}P < 0.01$, $^{***}P < 0.001$, $^{****}P < 0.0001$, data are mean ± SEM.

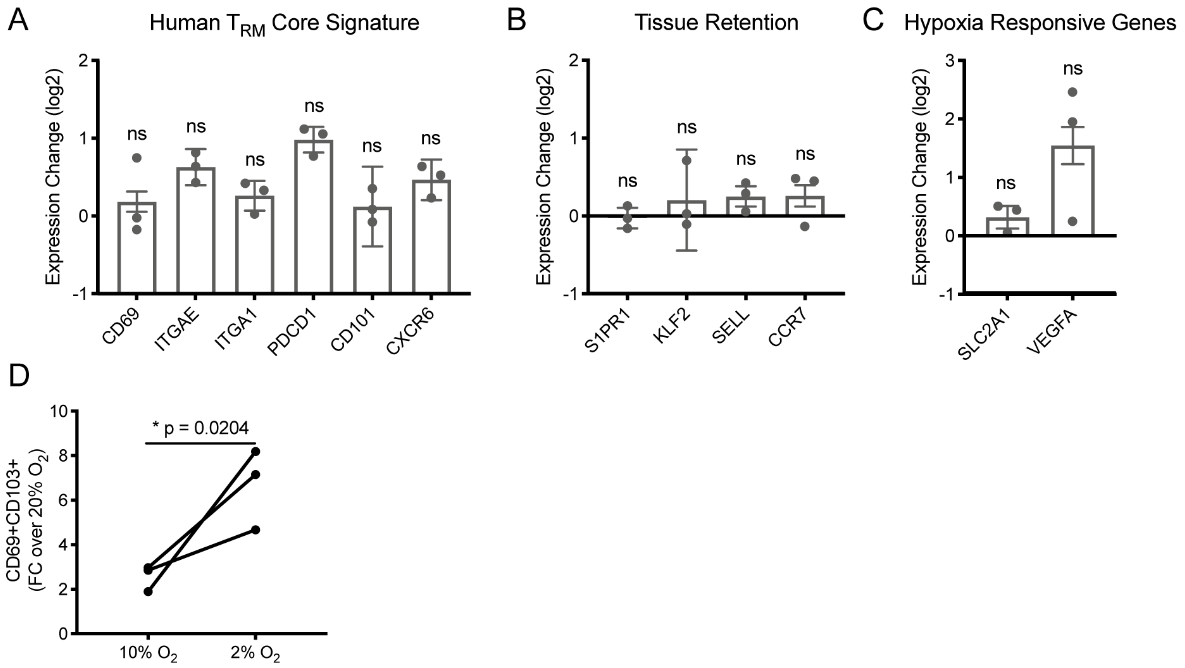

Supplemental Figure 2. Human CD8+ T-cells differentiated in 10% O_2 and TGF-β1 do not have a T_{RM}-like transcriptional profile. Naïve CD8+ T-cells isolated from PBMCs were activated in 20% O_2 (AtmosO_2) or 10% O_2 (circulationO_2) for 4 days and then for an additional 2 days with the addition of rhTGF-β1. Expression levels of T_{RM}-associated genes were analyzed via quantitative real-time PCR. (**A**-**C**) Fold change of gene transcript levels in 10% O_2 + TGF-β1 over 20% O_2 + TGF-β1. (**D**) The frequency of the CD69+CD103+ T_{RM}-like population was assessed by flow cytometry and compared to cultures activated in 2% O_2 (with TGF-β1 on day 4) by normalization to parallel cultures at 20% O_2. *n* = 3, 2 independent experiments; (**A**-**C**) Paired t-test with Benjamini, Krieger and Yekutieli correction for multiple comparisons; FDR < 0.05, data are mean ± SEM; (**D**) unpaired t-test, *$P < 0.05$.

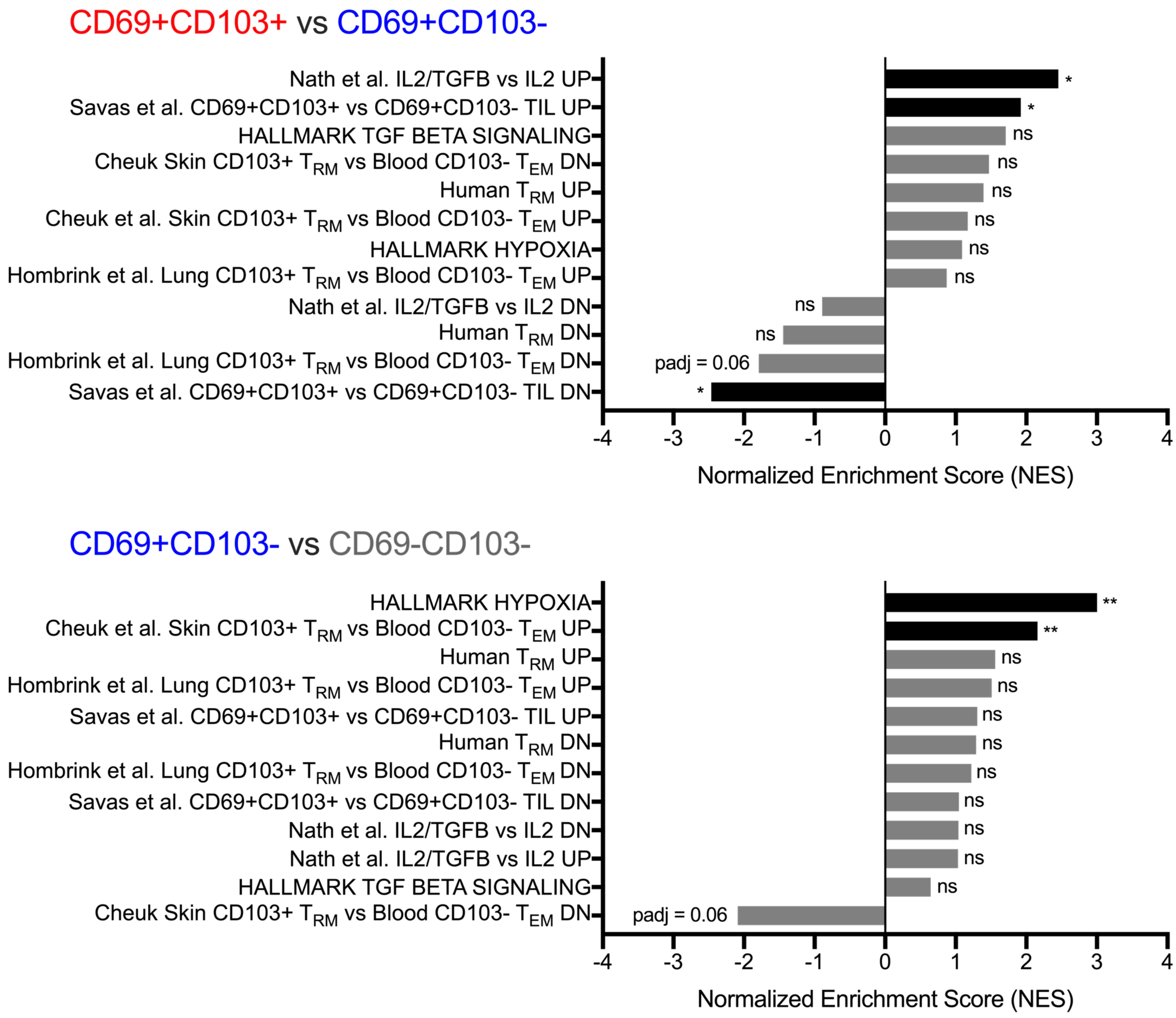

Supplemental Figure 3. GSEA of cells differentiated in hypoxia + TGF-β1 (CD69+CD103+) and hypoxia alone (CD69+CD103-). CD69-CD103- (20% O_2), CD69+CD103- (2% O_2), and CD69+CD103+ (2% O_2 + TGF-β1) CD8+ T-cells were generated as described earlier and sorted before RNA isolation and transcriptome analysis via RNA-sequencing (*n* = 3). GSEA of relevant gene signatures derived from endogenous T_{RM} and TIL_{RM}, presented as normalized enrichment score (NES), **padj* < 0.05, ***padj* < 0.01.

A

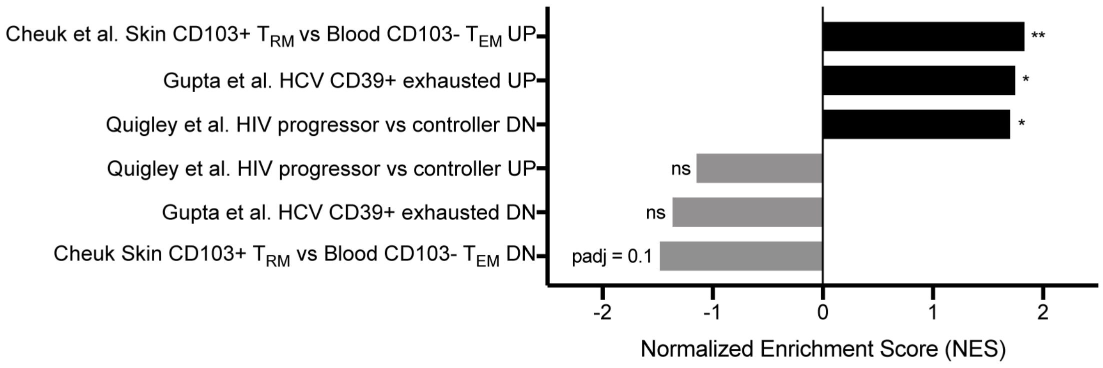

B

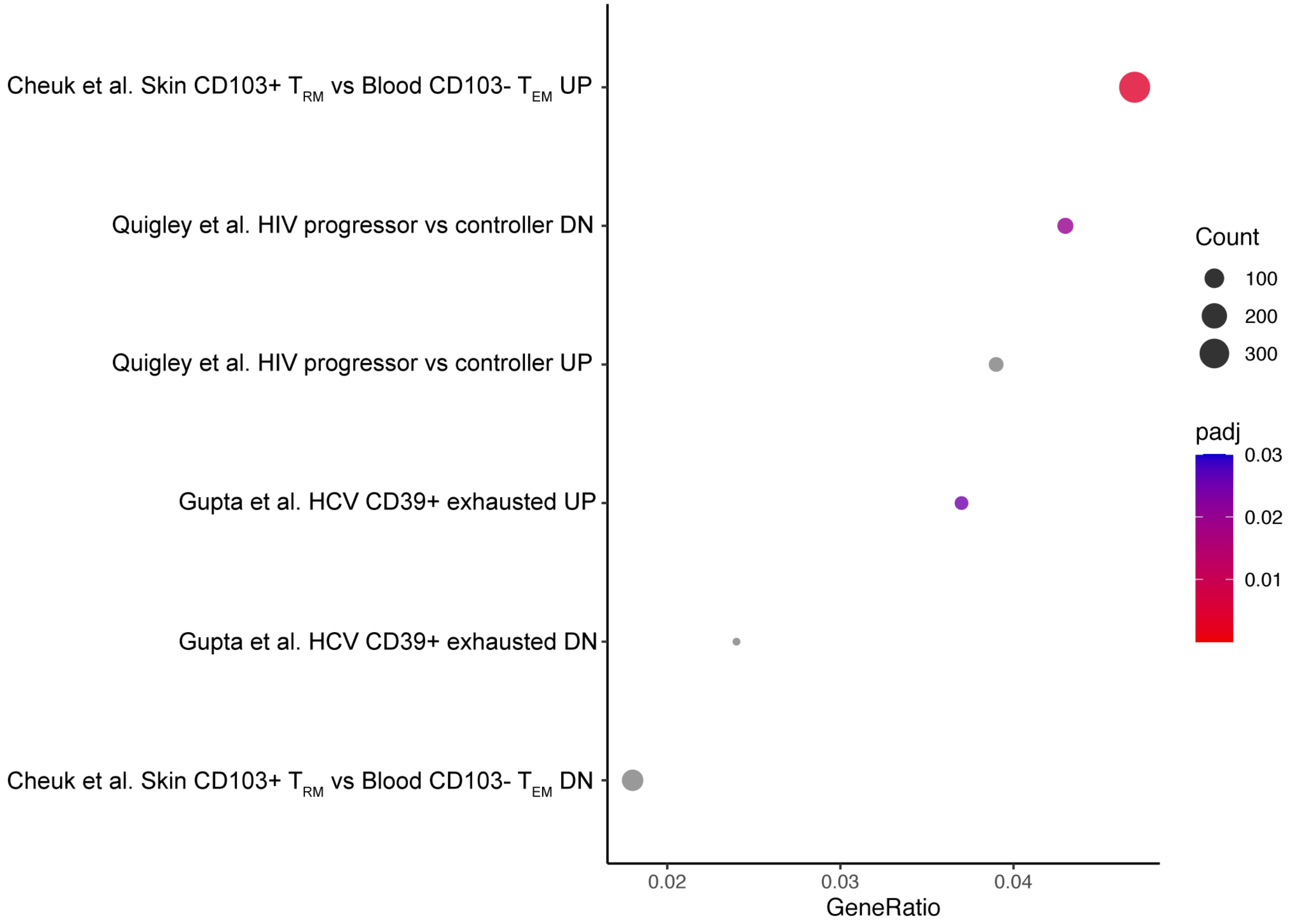

Supplemental Figure 4. Hypoxia and TGF-β1 induced T_{RM}-phenotype cells do not exhibit an exhaustion signature. CD69-CD103- (20% O_2) and CD69+CD103+ (2% O_2 + TGF-β1) CD8+ T-cells were generated as described earlier and sorted before RNA isolation and transcriptome analysis via RNA-sequencing (n = 3). (**A** and **B**) GSEA of published human T-cell exhaustion signatures in the transcriptome of CD69+CD103+ vs. CD69-CD103-, presented as normalized enrichment score, *$padj$ < 0.05, **$padj$ < 0.01. (**B**) Count refers to the number of enriched genes, GeneRatio refers to the ratio of enriched genes to the total geneset.

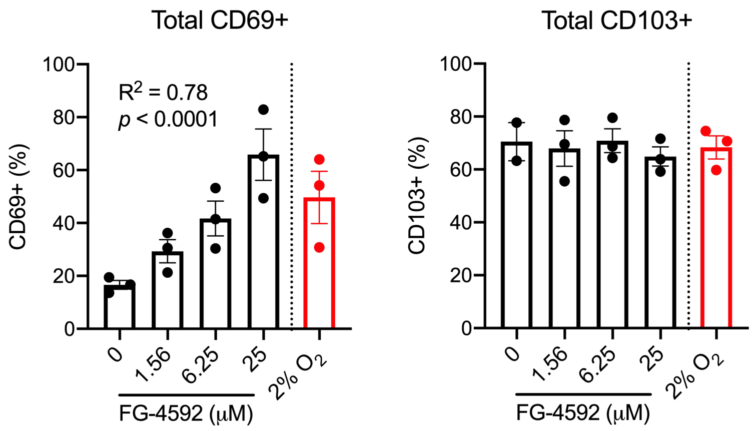

Supplemental Figure 5. HIF stabilization primarily drives CD69 expression. Naïve CD8+ T-cells were activated in 20% O_2 (AtmosO_2) in the presence of FG-4592 for 4 days and then for an additional 2 days with TGF-β1. Cells activated in 2% O_2 with addition of TGF-β1 on day 4 are shown in red for comparison. Frequency of the total CD69+ population and total CD103+ population was assessed by flow cytometry. *n* = 3, one-way ANOVA followed by test for trend, data are mean ± SEM.
